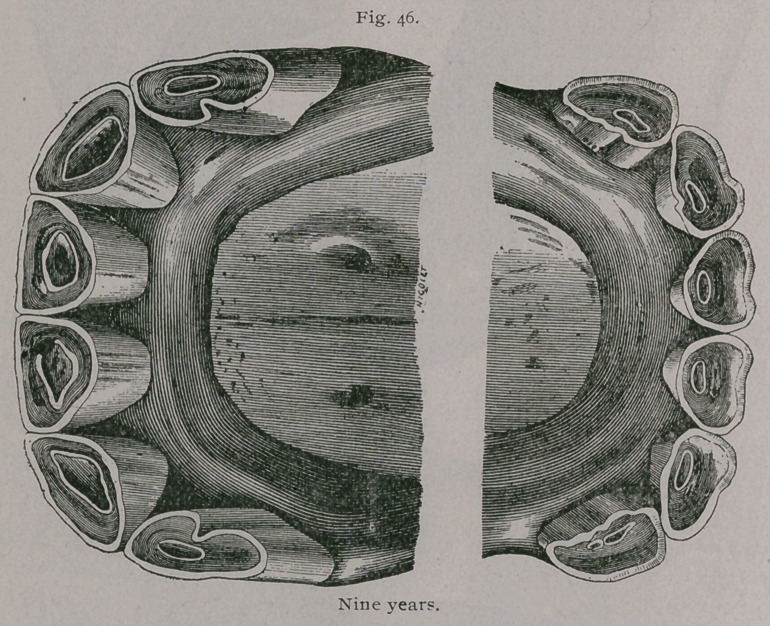# Age of the Horse, Ox, Dog, and Other Domesticated Animals

**Published:** 1890-11

**Authors:** R. S. Huidekoper

**Affiliations:** Veterinarian


### AGE OF THE HORSE OX, DOG AND OTHER DOMESTICATED ANIMALS.

By R. S. Huidekoper, M. D., Veterinarian.

*Continued from page 582.*

*Eight years.*—The direction of the incisors is decidedly changed; the inferior and superior arches are opposed obliquely; seen from in front the teeth project at the line of apposition. In the profile this is more apparent, and the arch assumes more the form of an "ogive." The incisive arches are still regular but decidedly smaller than the earlier ages. All of the inferior tables are leveled; the pinchers and intermediate teeth are oval;

Fig. 45.

Fig. 45.

the corner teeth to become so. The cups commence to assume an angular form behind, and are narrow. The dental star has appeared in the pinchers and commences to show in the intermediate teeth between the anterior border of the table and the corresponding part of the cup.

Fig 46.

Fig. 46.

Nine years.

*Nine years.*—There is no special change to be seen from in front or proinfile, although ordinarily the teeth are more obliquely formed, less fresh looking than at eight years of age. The notch on the superior corner has generally disappeared; the tables, however, at this age are characteristic; the pinchers are rounded and their cups have assumed a triangular form; the dental star is narrower and more distinct and is nearly in the centre of the table. The intermediate teeth commence to become round, and the corner teeth are oval; the superior pincher teeth are sometimes leveled; the inferior incisive arch is narrower and depressed in the centre.

[TO BE CONTINUED.]

## Figures and Tables

**Fig. 45. f1:**
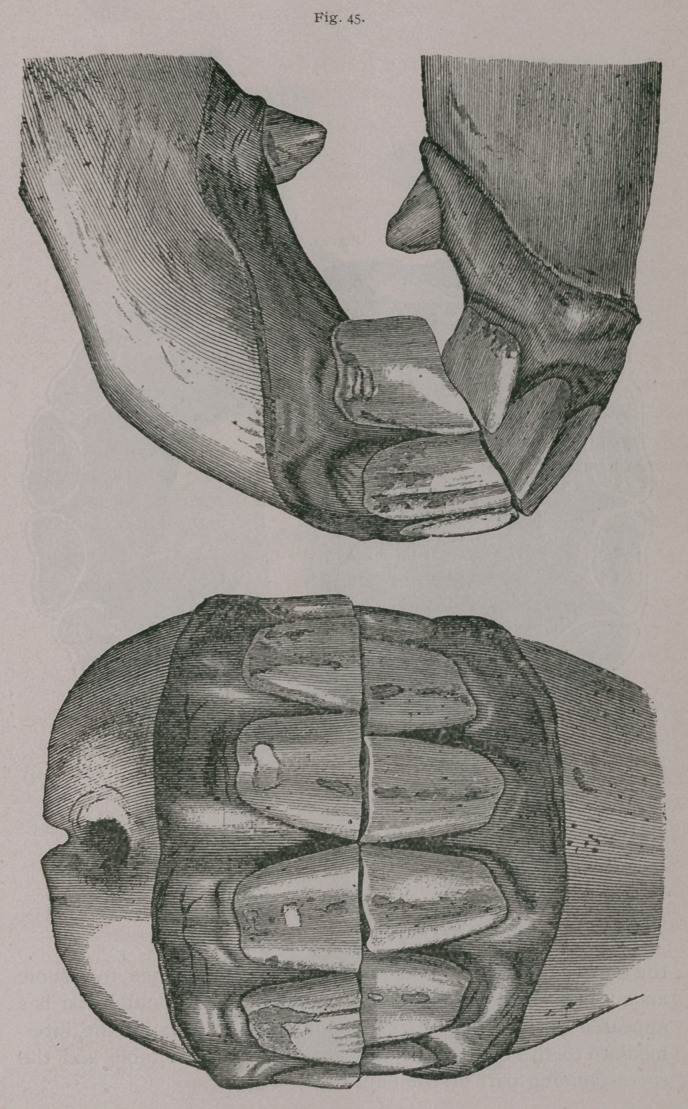


**Fig. 45. f2:**
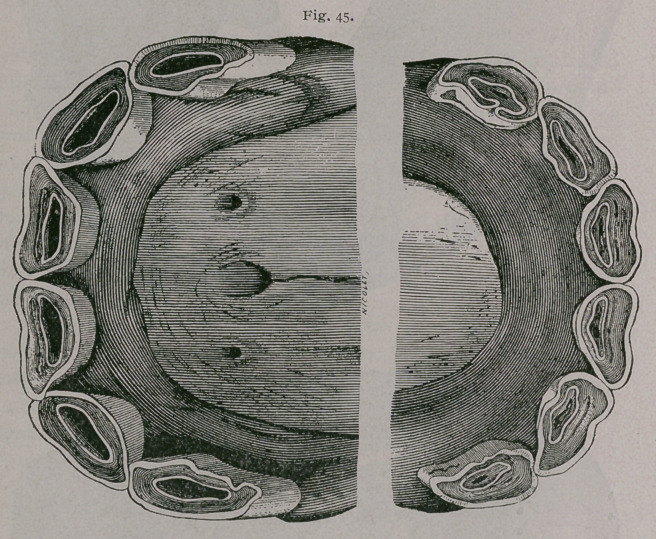


**Fig. 46. f3:**
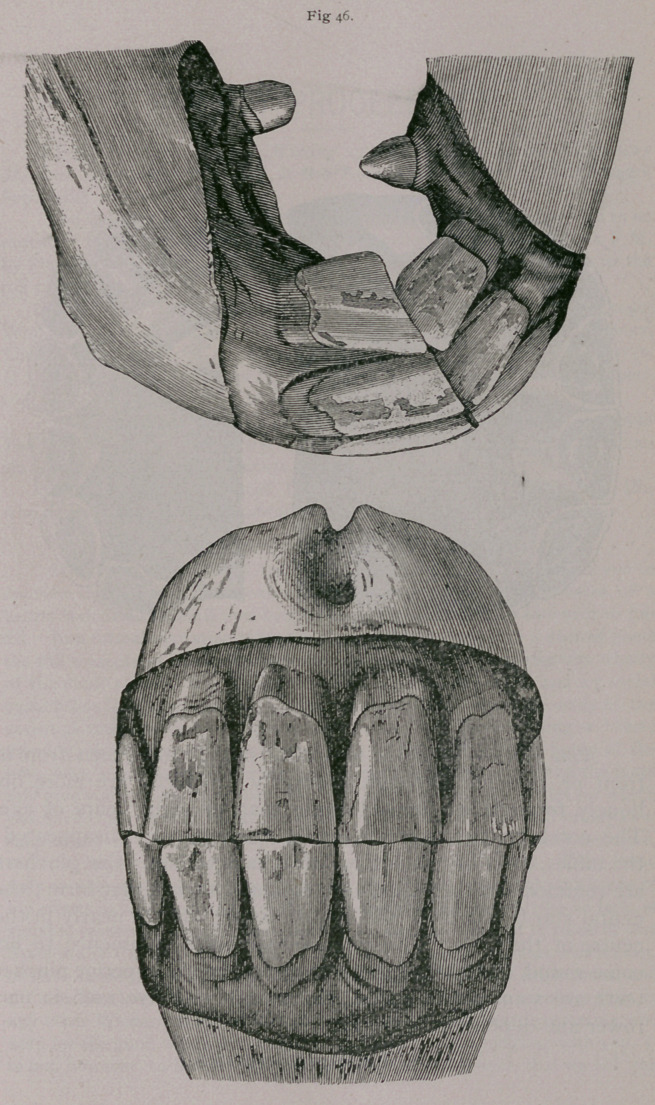


**Fig. 46. f4:**