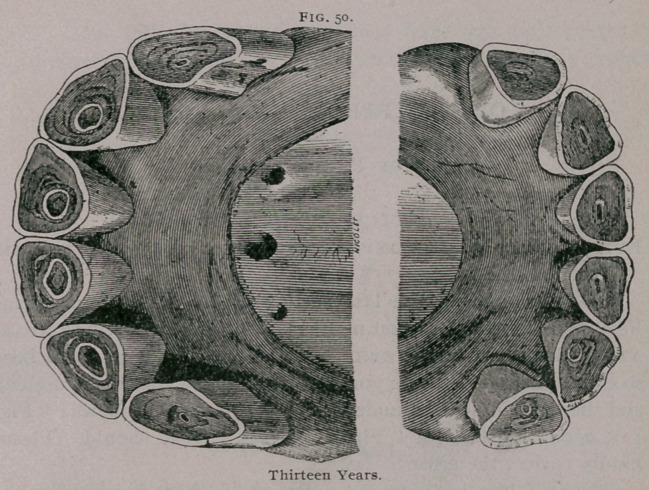# Erratum

**Published:** 1890-12

**Authors:** 


ERRATUM.

Nine Years.

On page 612, November number of the JOURNAL, the tables for eleven years were used instead of those of nine years as here given.

## Figures and Tables

**Figure f1:**